# Validity of European-centric cardiometabolic polygenic scores in multi-ancestry populations

**DOI:** 10.1038/s41431-023-01517-3

**Published:** 2024-01-05

**Authors:** Constantin-Cristian Topriceanu, Nish Chaturvedi, Rohini Mathur, Victoria Garfield

**Affiliations:** 1 Department of Population Science and Experimental Medicine, Institute of Cardiovascular Science, University College London, Gower Street, London, WC1E 6BT UK; 2 MRC Unit for Lifelong Health and Ageing, University College London, 1-19 Torrington Place, London, WC1E 7HB UK; 3 Centre for Primary Care, Wolfson Institute of Population Health, Queen Mary University of London, London, UK

**Keywords:** Risk factors, Disease prevention

## Abstract

Polygenic scores (PGSs) provide an individual level estimate of genetic risk for any given disease. Since most PGSs have been derived from genome wide association studies (GWASs) conducted in populations of White European ancestry, their validity in other ancestry groups remains unconfirmed. This is especially relevant for cardiometabolic diseases which are known to disproportionately affect people of non-European ancestry. Thus, we aimed to evaluate the performance of PGSs for glycaemic traits (glycated haemoglobin, and type 1 and type 2 diabetes mellitus), cardiometabolic risk factors (body mass index, hypertension, high- and low-density lipoproteins, and total cholesterol and triglycerides) and cardiovascular diseases (including stroke and coronary artery disease) in people of White European, South Asian, and African Caribbean ethnicity in the UK Biobank. Whilst PGSs incorporated some GWAS data from multi-ethnic populations, the vast majority originated from White Europeans. For most outcomes, PGSs derived mostly from European populations had an overall better performance in White Europeans compared to South Asians and African Caribbeans. Thus, multi-ancestry GWAS data are needed to derive ancestry stratified PGSs to tackle health inequalities.

## Introduction

A polygenic score (PGS) provides a personalised estimate of an individual’s genetic liability to a disease. These are calculated as weighted sums of single nucleotide polymorphisms (SNPs) [1]. Because most existing PGSs have been derived from genome wide association studies (GWASs) conducted in populations of European ancestry [2, 3], their validity in other ancestry groups remains unconfirmed. Therefore, although PGSs are an exciting prospect for precision medicine, they have the potential to perpetuate or widen existing health inequalities if they lead to invalid or misleading inference of disease risk in non-European populations.

In genetic studies, genetic ancestry is commonly used as a proxy for the social construct of ethnicity (and vice versa). However, ethnicity is a complex concept which includes genetic ancestry and a wide range of social constructs (e.g., cultural practices, health beliefs, language, religion, and self-identification) amongst others [4]. In general, genetic ancestry is thought to better reflect genetic relatedness than ethnicity, due to the fact that ethnicity is a broader social concept which incorporates a wide variety of environmental measures such as socioeconomic status and lifestyle [5]. However, there is considerable overlap between genetic ancestry and self-reported ethnicity, although ancestry does not capture the entirety of an individual’s ethnic identity [6]. Thus, self-reported ethnicity is important when examining health disparities related to the wider socio-cultural and environmental determinants of health in addition to biological and genetic factors [5].

PGSs derived in European ancestry populations generally transfer less well to African [7] or South Asian [8] populations. However, studies reporting the transferability to Hispanics have reported conflicting results [9, 10]. Even then, within ancestry heterogeneity can contribute to different predictive powers in ethnic sub-groups. For example, amongst Hispanics, the PGSs can have different performances based on ancestry clusters [11]. Thus, the transethnic transferability of PGSs remains a matter of debate.

Worldwide, there are 500–700 million individuals with diabetes mellitus (DM), 90% of whom have type 2 DM (T2DM) [12]. The prevalence of T2DM differs by age (more common in older people), sex (more common in men) and ethnicity. In the United Kingdom, South Asians are more likely to suffer from diabetes [13], followed by those from an African Caribbean background [14], both of whom have 2–3-fold higher risk of developing T2DM compared to White groups with onset almost a decade earlier [15]. In addition, South Asians and African Caribbeans are more likely to have higher serum glycated haemoblobin A1c (HbA1c) levels even in the absence of diabetes [16] and poorer glycaemic control in established diabetes [17].

In addition to ethnic differences in diabetes risk, cardiovascular diseases (CVDs) also vary across ethnicities. Compared to White Europeans, South Asians are more likely to develop CVD (i.e., coronary artery disease [CAD] and stroke), whilst those from an African Caribbean background are more likely to suffer from stroke [18]. Cardiovascular risk factors generally map to these ethnic differences in CVD outcomes. African Caribbeans generally have healthier lipid profiles (e.g., higher high-density lipoproteins [HDL] and lower total triglycerides [TTG] [19]) compared to White Europeans and South Asians, in whom lipoprotein profiles are most adverse [20]. In contrast, hypertension is more frequent in African Caribbeans than White Europeans [21]. The picture is more complex for South Asians, who have an equivalent or lower blood pressure (BP) than Europeans at younger ages [22], but subsequently experience a steeper BP trajectory resulting in higher later life BP [23].

Whether PGSs derived mostly from White ancestry GWAS data can capture differences by self-reported ethnicity in cardiometabolic traits  remains unclear. Using data from the UK Biobank (UKB), this study aimed to explore the prognostic value of transethnic transferability for a wide range of cardiometabolic PGSs and their respective observed outcomes. Our focus was on participants of South Asian and African Caribbean ethnicity in relation to White Europeans as these are the largest ethnic minority groups in the UK and are therefore well represented in UKB.

## Methods

### Study population

The UKB is a large UK based prospective cohort study with >500,000 participants recruited between 2006 and 2010 when study participants were aged 40–69 years old, and features demographic, genetic, health outcomes and imaging data for its participants [24]. We used the self-reported ethnicity variable which was defined according to the 2001 UK Census guidelines. The breakdown of self-reported ethnicity in UKB is 94.4% White Europeans, 0.2% South Asians, 0.2% African Caribbeans, and 5.2% other/unknown. Ancestry was previously derived in the UK Biobank using principal component analysis (PCA) and clustering, and it shows a good agreement with the self-reported ethnicity [25] we are using in this study.

### Polygenic scores

We used the standard and enhanced PGSs derived by Thompson et al., for which methodology has been previously described in detail [26]. Standard UKB PGSs contain only external GWAS data, whilst the enhanced UKB PGSs contain in addition UKB GWAS data. In December 2022, we selected the available standard and enhanced cardiometabolic UKB PGSs   namely: (1) type 1 DM (T1DM), (2) T2DM, (3) HbA1c, (4) body mass index (BMI), (4) hypertension, (5) CAD, (6) ischaemic stroke, (7) CVD, (8) HDL, (9) low-density lipoproteins (LDL), (10) total cholesterol, and (11) TTG. For total cholesterol and triglycerides, only an enhanced PGSs was available.

To derive the standard PGSs, Thompson et al. [26] conducted a literature review to identify GWAS summary statistics from external studies. These included: Atherosclerosis Risk in Communities (ARIC); Discovery, Biology and Risk of Inherited Variants in Breast Cancer (DRIVE); Electronic Medical Records and Genomics (eMERGE); BioMe BioBank; Jackson Heart Study (JHS); Multi-Ethnic Cohort (MEC); Multi-Ethnic Study of Atherosclerosis (MESA); Omics in Lations (OLA); and GWAS for Breast Cancer in the African Diaspora (ROOT study). To derive the enhanced PGSs, Thompson et al. [26] used a custom Axiom genotyping array (able to assay 825,927 genetic variants) followed by genome-wide imputation. Then, UKB GWAS summary statistics for each trait were obtained using logistic regression for binary outcomes, and linear regression for continuous outcomes, adjusting for age, sex, genotyping chip, and ancestry principal components (PCs). GWAS data were then combined using a Bayesian fixed-effects inverse variance meta-analysis model. UKB and external GWAS data were meta-analysed to yield the enhanced PGSs, whilst external GWAS data without UKB data were combined to obtain the standard PGSs. Both the standard and enhanced PGSs were derived in 70% of the dataset and tested in the remaining 30% to avoid overfitting. Genetic ancestry classification was done using the same methodology which showed a good overlap between self-reported ethnicity and genetic ancestry [25]. The proportion of the genotypes associated with White Europeans, South Asians and African Caribbeans ancestry was determined using a subset of common SNPs from the 1000 Genomes reference dataset, and genetic PCA was conducted to derive the centroid coordinates for ancestry groups, and to further define the ancestry categories [25]. The PGSs were then centred by subtracting out the PGS value predicted from a linear regression of the PGS against the first 4 PCs fitted in the 1000 Genomes Project individuals [27]. Lastly, the centred PGS was divided by the standard deviation (SD) in the corresponding ancestry group. The focus of our work are the enhanced PGSs as these have been shown by Thompson et al. [26] to have a higher predictive performance.

### Cardiometabolic outcomes

All outcomes were evaluated using information captured at the baseline assessment between 2006 and 2010 in the 22 recruitment centres across England, Scotland, and Wales. These included the presence of T1DM (yes/no), T2DM (yes/no), HbA1c (mmol/mol), BMI (kg/m^2^), hypertension (yes/no), CAD (yes/no), stroke (yes/no), CVD (yes/no), HDL (mmol/l), LDL (mmol/l), total cholesterol (mmol/l) and TTG (mmol/l). T1DM and T2DM were defined using an algorithm which was validated against primary care records, taking into account the self-report, doctor diagnosis and the use of diabetes medications [28]. BMI (kg/m^2^) was calculated as the ratio of weight to height^2^. The presence of hypertension at baseline was defined as either: (1) self-report of anti-hypertensive medication use, (2) systolic BP > 140 mmHg or (3) diastolic BP > 80 mmHg. The presence of CAD, stroke and CVD (i.e., CAD + stroke) were based on the baseline self-report, nursing interview and linked inpatient hospital data as previously described [29]. We did not use incident data as there are known healthcare access disparities among ethnic groups in the UK which could introduce bias [30]. HbA1c, HDL, LDL, total cholesterol and TTG were quantified from the baseline blood samples [25].

### Covariates

Sex was self-reported as male or female, and age (years) was recorded at the time of recruitment. Area based Townsend deprivation scores were used to capture socio-economic position (SEP) [31]. The primary care survey provided data on the prescribed medications of each study participant.

### Statistical analysis

All analyses were performed in R 4.2.1 [32]. Data distributions were assessed using histograms. Continuous variables were expressed as mean ± 1 SD or median (interquartile range) as appropriate; categorical variables were expressed as counts and percentages.

Participants were categorised based on self-reported ethnicity as White European, South Asian, and African Caribbean. Individuals of mixed, other, and unknown ethnicity were not included due to small sample sizes. All analyses were conducted within each ethnic group. We used the PGSs as the  indipendent variables to test their association with their corresponding cardiometabolic outcomes. For binary outcomes, generalised linear models (glms) with binominal distribution (i.e., logistic regression) were employed. The continuous outcomes were either slightly or heavily skewed. For example, BMI had a skewness greater than 1, while HbA1c and TTG a skewness exceeding 2. As the gamma distribution can flexibly accommodate positively skewed data due to its shape and scale parameters, we used glms with gamma distribution and identity link for our continuous outcomes.

Two regression models were compared. Model 1 was unadjusted to obtain raw estimates. For all outcomes, model 2 was adjusted for age, sex, and SEP in order to obtain more accurate and precise regression estimates. As adjustment for genetic PCs was previously used to control for ancestry during the PGS derivation process, further adjustment for PCs was not pursued. Since this study did not attempt to explore mechanistic pathways downstream of the genotype but upstream of the phenotypes, further models with adjustment for mediators were not pursued. Model assumptions were verified with regression diagnostics and found to be satisfied. Results were then corrected for multiple testing using a false discovery rate of 0.05 [33].

Using a 30% testing dataset, the classification performance (i.e., predicting the binary outcome) of both logistic regression models were evaluated using the receiver operating characteristic (ROC) curve. The area under the  curve (AUC) and its associated 95% confidence interval (CI) was derived for each ethnicity for each binary outcome. The ROC AUCs were compared between ethnicities using DeLong’s test. For continuous outcomes, we compared the effect sizes derived from the glms which capture the increase  in outcome per unit increase in the PGSs. Since the PGSs underwent a PC-based ancestry centring and had a normal distribution with a similar SD of approximatively 1 (Table 1), the effect sizes was not further standardised.

**Table 1:** Participant characteristics per ethnic group.

	White European [1]		South Asian [2]		African Caribbean [3]		[1] vs [2]	[1] vs [3]	[2] vs [3]
	*n*	Count (%) or Mean ± sd	*n*	Count (%) or Mean ± sd	*n*	Count (%) or Mean ± sd	*p*-value*	*p*-value**	*p*-value***
Polygenic scores									
T1DM	457611	0.05 ± 1.13	7644	−0.10 ± 1.03	7621	−0.01 ± 1.11	**<0.0001**	**<0.0001**	**<0.0001**
T2DM	457611	−0.15 ± 1.00	7644	0.04 ± 1.02	7621	0.04 ± 1.13	**<0.0001**	**<0.0001**	0.588
HbA1c	457611	0.09 ± 1.07	7644	0.06 ± 1.02	7621	0.00 ± 1.10	**0.009**	**<0.0001**	**0.001**
BMI	457611	−0.21 ± 1.02	7644	0.04 ± 1.03	7621	−0.03 ± 1.06	**<0.0001**	**<0.0001**	**<0.0001**
Hypertension	457611	−0.04 ± 0.99	7644	−0.16 ± 1.03	7621	−0.19 ± 1.06	**<0.0001**	**<0.0001**	0.167
CVD	457611	−0.11 ± 1.01	7644	0.11 ± 1.05	7621	−0.13 ± 1.14	**<0.0001**	0.112	**<0.0001**
CAD	457611	−0.17 ± 0.99	7644	0.06 ± 1.07	7621	−0.19 ± 1.15	**<0.0001**	0.146	**<0.0001**
Stroke	457611	−0.02 ± 0.97	7644	−0.02 ± 1.02	7621	−0.22 ± 1.10	0.650	**<0.0001**	**<0.0001**
HDL	457611	0.01 ± 1.06	7644	0.10 ± 1.02	7621	−0.01 ± 1.04	**<0.0001**	0.092	**<0.0001**
LDL	457611	−0.07 ± 1.05	7644	0.11 ± 1.02	7621	−0.04 ± 1.13	**<0.0001**	**0.0002**	**<0.0001**
Outcomes									
T1DM, yes (%)	472036	1697 (0.36%)	8052	35 (0.43%)	8048	26 (0.32%)	0.307	0.654	0.306
T2DM, yes (%)	472036	20494 (4.34%)	8052	1346 (16.72%)	8048	850 (10.56%)	**<0.0001**	**<0.0001**	**<0.0001**
HbA1c, mmol/mol	441777	35.99 ± 6.45	7300	40.83 ± 10.57	6268	39.43 ± 10.10	**<0.0001**	**<0.0001**	**<0.0001**
BMI, kg/m^2^	469759	27.40 ± 4.78	7814	27.29 ± 4.47	7897	29.53 ± 5.38	**0.027**	**<0.0001**	**<0.0001**
Hypertension, yes (%)	472036	305,714 (64.76%)	8052	5493 (68.22%)	7048	5824 (72.59%)	**<0.0001**	**<0.0001**	**<0.0001**
CVD, yes (%)	471959	32,539 (6.89%)	8051	851 (10.12%)	8047	431 (5.36%)	**<0.0001**	**<0.0001**	**<0.0001**
CAD, yes (%)	472036	21,278 (4.51%)	8052	599 (7.44%)	8048	260 (3.23%)	**<0.0001**	**<0.0001**	**<0.0001**
Stroke, yes (%)	471217	7259 (1.54%)	7908	132 (1.67%)	7968	126 (1.58%)	0.382	0.804	0.708
HDL, mmol/l	404554	1.41 ± 0.52	6748	1.20 ± 0.41	6745	1.39 ± 0.51	**<0.0001**	**<0.0001**	**<0.0001**
LDL, mmol/l	441161	3.57 ± 0.92	7397	3.33 ± 0.9	7276	3.26 ± 0.88	**<0.0001**	**<0.0001**	**<0.0001**
Total cholesterol, mmol/l	441872	5.71 ± 1.18	7410	5.29 ± 1.15	7292	5.24 ± 1.13	**<0.0001**	**<0.0001**	**0.005**
TTG, mmol/l	441633	1.76 ± 1.04	7404	1.97 ± 1.18	7290	1.28 ± 0.75	**<0.0001**	**<0.0001**	**<0.0001**
Covariates									
Age, years	472036	56.76 ± 8.03	8052	53.4 ± 8.45	8048	51.94 ± 7.08	**<0.0001**	**<0.0001**	**<0.0001**
Sex, male (%)	472036	214,955 (45.54%)	8052	4304 (53.45%)	8048	3402 (42.27%)	**<0.0001**	**<0.0001**	**<0.0001**
SEP, Townsend deprivation index	472036	−1.45 ± 3.01	8043	0.24 ± 3.12	8028	2.64 ± 3.45	**<0.0001**	**<0.0001**	**<0.0001**

Continuous variables are presented in their corresponding units, whilst binary variables are presented as yes counts (percentages of yes counts). Of note, PGSs are unitless. Only the standard PGSs are shown here. The enhanced PGSs are presented in Supplementary Table S1.

*BMI* body mass index, *CAD* coronary artery disease, *CVD* cardiovascular disease, *HbA1c* glycated haemoglobin A1c, *HDL* high-density lipoproteins, *LDL* low-density lipoproteins*,* *PGS* polygenic score, *SEP* socio-economic position, *T1DM* type 1 diabetes mellitus, *T2DM* type 2 diabetes mellitus, *TTG* total triglycerides.

All *p*-values were derived using *t*-test for continuous variables and Chi-squared test for categorical ones. Significant *p*-values are highlighted in bold.

*White Europeans were compared with South Asians.

**White Europeans were compared with African Caribbeans.

***South Asians were compared with African Caribbeans.

### Sensitivity analyses

As a sensitivity analysis, model 2 was additionally adjusted for diabetes medications when HbA1c was the outcome and for lipid-lowering drugs when exploring HDL, LDL, total cholesterol and TTG as outcomes. PGSs are upstream of the cardiometabolic outcomes which are upstream of the medications (i.e., a causal chain). In instances where the medications can then affect back the cardiometabolic outcome (e.g., diabetes medications lowering HbA1c), adjusting for them allows the estimation of the direct association between the PGS and the cardiometabolic outcome. This adjustment esentially controls for unmeasured confounders downstream of the medication (e.g., access to healthcare, healthcare seeking behaviour etc.).

In addition, we also calculated the area under the precision-recall curve (PR-AUC) as the ROC AUC can be misleading when the outcomes are rare.

## Results

In this study we included 472,036 participants their characteristics and standard PGSs stratified by ethnicity are presented in Table 1, while their enhanced PGSs are presented in Supplementary Table S1. On average, both South Asians (53.4 years) and African Caribbeans (51.9 years) were younger than White Europeans (56.8 years) at the time of outcome assessment. Men comprised 45.5% of White, 54.5% of South Asians and 42.3% of African Caribbeans. There were 45.7% South Asians, 70.5% African Caribbeans, and 23.2% White Europeans in the lowest quartile of the Townsend deprivation index. South Asians had the highest prevalence of T2DM (16.7%), CVD (10.1%), and CAD (7.4%), whilst African Caribbeans had the highest average BMI (29.5) and the highest prevalence of hypertension (72.6%). Despite the PC-based ancestry centring, the PGSs experienced small residual deviations from absolute zero in South Asians and African Caribbeans (Table 1). Model 1 and model 2 results for the standard and enhanced PGSs are presented in Table 2. Results from model 2 for the enhanced PGSs are presented below.

**Table 2:** Regression results stratified per ethnicity.

			White European			South Asian			African Caribbean		
Outcome	PGS type	Model	*n*	β or OR (95% CI)	*p*-value	*n*	β or OR (95% CI)	*p*-value	*n*	β or OR (95% CI)	*p*-value
T1DM	Standard	Model 1	457,611	2.85 (2.75, 2.96)	**<0.0001**	7644	1.49 (1.08, 2.01)	**0.013**	7621	1.35 (0.96, 1.87)	0.080
		Model 2	457,072	2.87 (2.76, 2.98)	**<0.0001**	7635	1.50 (1.09, 2.04)	**0.011**	7604	1.36 (0.96, 1.89)	0.764
	Enhanced	Model 1	76,877	3.05 (2.78, 3.35)	**<0.0001**	7637	1.51 (1.10, 2.05)	**0.009**	7618	1.39 (0.99, 1.93)	0.053
		Model 2	76,793	3.09 (2.72, 3.41)	**<0.0001**	7627	1.52 (1.11, 2.07)	**0.008**	7601	1.40 (0.99, 1.95)	0.520
T2DM	Standard	Model 1	457,611	2.34 (2.31, 2.38)	**<0.0001**	7644	1.86 (1.74, 1.99)	**<0.0001**	7621	1.47 (1.38, 1.58)	**<0.0001**
		Model 2	457,072	2.42 (2.38, 2.46)	**<0.0001**	7635	2.00 (1.87, 2.15)	**<0.0001**	7604	1.53 (1.43, 1.64)	**<0.0001**
	Enhanced	Model 1	76,877	2.23 (2.15, 2.31)	**<0.0001**	7637	1.81 (1.70, 1.93)	**<0.0001**	7601	1.38 (1.30, 1.48)	**<0.0001**
		Model 2	76,793	2.48 (2.39, 2.58)	**<0.0001**	7628	2.05 (1.91, 2.20)	**<0.0001**	7601	1.51 (1.41, 1.62)	**<0.0001**
HbA1c	Standard	Model 1	437,520	0.84 (0.82, 0.85)	**<0.0001**	7187	1.32 (1.07, 1.56)	**<0.0001**	6214	0.48 (0.25, 0.72)	**<0.0001**
		Model 2	436,997	0.83 (0.81, 0.85)	**<0.0001**	7179	1.28 (1.04, 1.52)	**<0.0001**	6203	0.51 (0.28, 0.73)	**<0.0001**
	Enhanced	Model 1	73,455	1.69 (1.65, 1.73)	**<0.0001**	7181	1.82 (1.60, 2.05)	**<0.0001**	6211	1.07 (0.83, 1.30)	**<0.0001**
		Model 2	73,377	1.69 (1.65, 1.73)	**<0.0001**	7173	1.79 (1.57, 2.00)	**<0.0001**	6200	1.03 (0.81, 1.26)	**<0.0001**
BMI	Standard	Model 1	455,985	1.32 (1.31, 1.33)	**<0.0001**	7462	1.10 (1.00, 1.20)	**<0.0001**	7505	0.73 (0.61, 0.85)	**<0.0001**
		Model 2	455,453	1.31 (1.30, 1.32)	**<0.0001**	7453	1.08 (0.98, 1.18)	**<0.0001**	7488	0.73 (0.62, 0.85)	**<0.0001**
	Enhanced	Model 1	76,572	1.72 (1.69, 1.74)	**<0.0001**	7455	1.34 (1.24, 1.43)	**<0.0001**	7502	0.91 (0.80, 1.02)	**<0.0001**
		Model 2	76,490	1.71 (1.68, 1.74)	**<0.0001**	7446	1.31 (1.22, 1.40)	**<0.0001**	7485	0.90 (0.80, 1.00)	**<0.0001**
Hypertension	Standard	Model 1	457,611	1.52 (1.52, 1.54)	**<0.0001**	7644	1.46 (1.38, 1.53)	**<0.0001**	7621	1.19 (1.13, 1.25)	**<0.0001**
		Model 2	457,072	1.57 (1.56, 1.59)	**<0.0001**	7635	1.51 (1.43, 1.60)	**<0.0001**	7604	1.21 (1.15, 1.28)	**<0.0001**
	Enhanced	Model 1	76,877	1.63 (1.60, 1.65)	**<0.0001**	7637	1.54 (1.47, 1.63)	**<0.0001**	7618	1.28 (1.22, 1.34)	**<0.0001**
		Model 2	76,793	1.79 (1.76, 1.82)	**<0.0001**	7628	1.67 (1.58, 1.77)	**<0.0001**	7628	1.67 (1.58, 1.77)	**<0.0001**
CVD	Standard	Model 1	457,539	1.49 (1.47, 1.51)	**<0.0001**	7643	1.40 (1.30, 1.50)	**<0.0001**	7620	1.13 (1.04, 1.24)	**0.0007**
		Model 2	457,001	1.53 (1.52, 1.55)	**<0.0001**	7634	1.48 (1.37, 1.60)	**<0.0001**	7603	1.14 (1.04, 1.25)	**0.007**
	Enhanced	Model 1	76,868	1.56 (1.51, 1.60)	**<0.0001**	7636	1.47 (1.36, 1.59)	**<0.0001**	7617	1.20 (1.09, 1.32)	**0.002**
		Model 2	76,784	1.61 (1.56, 1.66)	**<0.0001**	7627	1.58 (1.45, 1.71)	**<0.0001**	7600	1.20 (1.09, 1.32)	**0.0002**
CAD	Standard	Model 1	457,611	1.71 (1.68, 1.73)	**<0.0001**	7644	1.48 (1.36, 1.61)	**<0.0001**	7621	1.18 (1.05, 1.32)	**0.004**
		Model 2	457,072	1.78 (1.75, 1.81)	**<0.0001**	7635	1.55 (1.42, 1.69)	**<0.0001**	7604	1.18 (1.06, 1.33)	**0.004**
	Enhanced	Model 1	76,877	1.83 (1.76, 1.90)	**<0.0001**	7637	1.60 (1.47, 1.74)	**<0.0001**	7618	1.21 (1.08, 1.36)	**0.001**
		Model 2	76,793	1.91 (1.84, 1.99)	**<0.0001**	7628	1.70 (1.55, 1.86)	**<0.0001**	7601	1.22 (1.08, 1.37)	**0.001**
Stroke	Standard	Model 1	456,848	1.34 (1.31, 1.38)	**<0.0001**	7514	1.34 (1.12, 1.61)	**0.001**	7545	1.13 (0.95, 1.35)	0.164
		Model 2	456,310	1.34 (1.30, 1.37)	**<0.0001**	7505	1.36 (1.14, 1.64)	**0.0008**	7528	1.13 (0.95, 1.35)	0.167
	Enhanced	Model 1	76,703	1.43 (1.34 1.52)	**<0.0001**	7507	1.39 (1.15, 1.67)	**0.0006**	7542	1.23 (1.05, 1.45)	**0.012**
		Model 2	76,619	1.42 (1.33, 1.51)	**<0.0001**	7498	1.42 (1.17, 1.71)	**0.0003**	7525	1.23 (1.04, 1.45)	**0.015**
HDL	Standard	Model 1	399,315	0.14 (0.14, 0.14)	**<0.0001**	6607	0.11 (0.10, 0.12)	**<0.0001**	6610	0.10 (0.09, 0.11)	**<0.0001**
		Model 2	396,414	0.13 (0.13, 0.14)	**<0.0001**	6373	0.11 (0.10, 0.12)	**<0.0001**	6431	0.10 (0.09, 0.11)	**<0.0001**
	Enhanced	Model 1	67,042	0.14 (0.14, 0.14)	**<0.0001**	6602	0.11 (0.10, 0.12)	**<0.0001**	6607	0.09 (0.08, 0.10)	**<0.0001**
		Model 2	66,465	0.14 (0.13, 0.14)	**<0.0001**	6368	0.11 (0.10, 0.11)	**<0.0001**	6428	0.09 (0,08, 0.10)	**<0.0001**
LDL	Standard	Model 1	435,509	0.24 (0.24, 0.24)	**<0.0001**	7240	0.16 (0.14, 0.18)	**<0.0001**	7135	0.15 (0.14, 0.17)	**<0.0001**
		Model 2	432,326	0.24 (0.24, 0.24)	**<0.0001**	6988	0.15 (0.13, 0.17)	**<0.0001**	6940	0.15 (0.14, 0.17)	**<0.0001**
	Enhanced	Model 1	73,124	0.27 (0.26, 0.27)	**<0.0001**	7234	0.18 (0.16, 0.20)	**<0.0001**	7132	0.22 (0.20, 0.23)	**<0.0001**
		Model 2	72,506	0.27 (0.26, 0.27)	**<0.0001**	6982	0.17 (0.15, 0.19)	**<0.0001**	6937	0.22 (0.20, 0.23)	**<0.0001**
Total cholesterol	Enhanced	Model 1	73,261	0.28 (0.27, 0.29)	**<0.0001**	7248	0.19 (0.17, 0.22)	**<0.0001**	7146	0.20 (0.18, 0.23)	**<0.0001**
		Model 2	72,640	0.28 (0.27, 0.29)	**<0.0001**	6996	0.18 (0.16, 0.21)	**<0.0001**	6951	0.20 (0.18, 0.23)	**<0.0001**
TTG	Enhanced	Model 1	73,203	0.24 (0.23, 0.25)	**<0.0001**	7243	0.28 (0.26, 0.30)	**<0.0001**	7144	0.09 (0.07, 0.11)	**<0.0001**
		Model 2	72,583	0.23 (0.22, 0.24)	**<0.0001**	6992	0.28 (0.26, 0.30)	**<0.0001**	6949	0.09 (0.07, 0.11)	**<0.0001**

For binary outcomes, logistic regressions were used to test for the associations between the PGSs as the independent variables and their corresponding cardiometabolic outcomes as the dependent variables. Effect sizes are presented in the form of ORs. For continuous outcomes, generalised linear models with gamma distribution and identity links were used instead and effect sizes are presented in the form of β regression coefficients. Model 1 was unadjusted to obtain crude estimates as the relationship between genotype and phenotype should be unconfounded. Model 2 was adjusted for age, sex, and SEP to obtain more accurate and precise effect size estimates. Significant *p*-values are highlighted in bold.

*β* regression coefficient, *CI* confidence interval, *OR* odds ratio. Other abbreviations as in Table 1.

### Type 1 diabetes

**Fig. 1:**
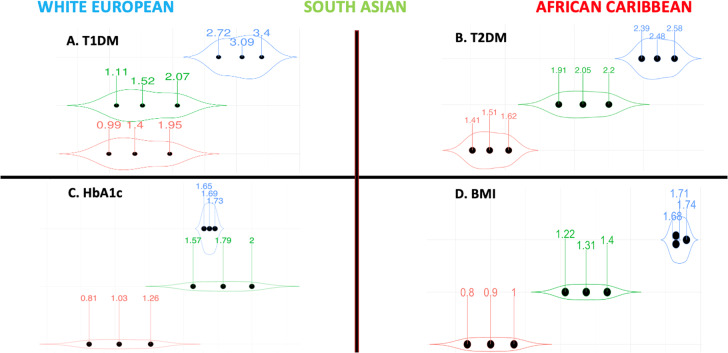
Violin plots highlighting the effect sizes per standard deviation increase in the enhanced PGSs for BMI and diabetes-related traits stratified by ethnicity. Our traits included: **(A)** T1DM, **(B)** T2DM, **(C)** HbA1c, and **(D)** BMI. For binary outcomes, logistic regressions were used to test for the associations between the PGSs as the independent variables and their corresponding cardiometabolic outcomes as the dependent variables. Effect sizes are presented in the form of ORs. For continuous outcomes, glms with gamma distribution were used instead and effect sizes are presented in the form of β regression coefficients. Models were adjusted for age, sex and SEP. In each violin plot, the middle dot represents the effect size, and the adjacent ones the lower and upper limits of the 95% CI. The violin shape reflects the wideness of the 95% CI. **Abbreviations**: BMI body mass index, CI confidence interval, glm generalised linear models, HbA1c glycated haemoglobinA1c, PGS polygenic score, OR odds ratio, SEP socio-economic position, T1DM type 1 diabetes mellitus, T2DM type 2 diabetes mellitus.

The association between the enhanced PGSs and T1DM was strongest for White Europeans (odds ratio [OR] 3.09 95% CI [2.72, 3.40]) followed by South Asians (OR 1.52 95% CI [1.11, 2.07]) and African Caribbeans (OR 1.40 95% CI [0.99, 1.95]) (Fig. 1A). The PGS’ predictive performance was highest in White Europeans (AUC 0.84 95% CI [0.80, 0.89]) followed by South Asians AUC 0.63 95% CI [0.49, 0.77] and African Caribbeans (AUC 0.50 95% CI [0.32,0.68]) (Table 3).

**Table 3:** Predictive power of PGSs for diabetes-related binary outcomes stratified by ancestry.

Outcome	PGS type	Model	White European [1]	South Asian [2]	African Caribbean [3]	[1] vs [2]	[1] vs [3]	[2] vs [3]
			AUC (95% CI)	AUC (95% CI)	AUC (95% CI)	*p*-value*	*p*-value**	*p*-value***
T1DM	Standard	Model 1	0.834 (0.814, 0.854)	0.683 (0.546, 0.820)	0.694 (0.449, 0.939)	**0.031**	0.265	0.933
		Model 2	0.843 (0.824, 0.862)	0.633 (0.482, 0.764)	0.513 (0.330, 0.697)	**0.003**	**0.0001**	0.329
	Enhanced	Model 1	0.844 (0.792, 0.889)	0.685 (0.550, 0.820)	0.709 (0.459, 0.969)	**0.029**	0.297	0.869
		Model 2	0.844 (0.800, 0.890)	0.628 (0.489, 0.769)	0.503 (0.322, 0.684)	**0.004**	**<0.0001**	0.246
T2DM	Standard	Model 1	0.719 (0.711, 0.724)	0.673 (0.644, 0.703)	0.645 (0.607, 0.683)	**0.004**	**0.0002**	0.250
		Model 2	0.786 (0.780, 0.792)	0.752 (0.726, 0.777)	0.734 (0.701, 0.767)	**0.010**	**0.003**	0.419
	Enhanced	Model 1	0.740 (0.724, 0.755)	0.681 (0.652, 0.710)	0.624 (0.585, 0.662)	**0.0005**	**<0.0001**	**0.021**
		Model 2	0.803 (0.790, 0.816)	0.756 (0.730, 0.781)	0.727 (0.693, 0.762)	**0.001**	**<0.0001**	0.197
Hypertension	Standard	Model 1	0.609 (0.606, 0.612)	0.582 (0.557, 0.607)	0.545 (0.520, 0.573)	**0.036**	**<0.0001**	0.055
		Model 2	0.686 (0.683, 0.689)	0.696 (0.673, 0.719)	0.689 (0.665, 0.712)	0.409	0.842	0.662
	Enhanced	Model 1	0.641 (0.634, 0.649)	0.601 (0.576, 0.626)	0.570 (0.544, 0.597)	**0.002**	**<0.0001**	0.093
		Model 2	0.705 (0.698, 0.712)	0.706 (0.683, 0.729)	0.695 (0.671, 0.718)	0.927	0.415	0.501
CVD	Standard	Model 1	0.603 (0.597, 0.609)	0.580 (0.541, 0.620)	0.548 (0.481, 0.594)	0.259	**0.024**	0.226
		Model 2	0.762 (0.757, 0.766)	0.731 (0.697, 0.765)	0.659 (0.607, 0.711)	0.081	**0.0001**	**0.023**
	Enhanced	Model 1	0.621 (0.607, 0.636)	0.591 (0.551, 0.631)	0.554 (0.497, 0.610)	0.162	**0.023**	0.293
		Model 2	0.767 (0.755, 0.779)	0.735 (0.7, 0.769)	0.664 (0.612, 0.716)	0.079	**0.0001**	**0.025**
CAD	Standard	Model 1	0.631 (0.624, 0.638)	0.601 (0.554, 0.648)	0.564 (0.488, 0.640)	0.222	0.088	0.418
		Model 2	0.789 (0.784, 0.795)	0.744 (0.707, 0.781)	0.696 (0.631, 0.761)	**0.017**	**0.005**	0.207
	Enhanced	Model 1	0.667 (0.651, 0.684)	0.626 (0.580, 0.673)	0.567 (0.495, 0.645)	0.103	**0.013**	0.211
		Model 2	0.805 (0.792, 0.818)	0.753 (0.716, 0.790)	0.699 (0.634, 0.764)	**0.010**	**0.002**	0.153
Stroke	Standard	Model 1	0.578 (0.565, 0.590)	0.520 (0.440, 0.600)	0.520 (0.428, 0.612)	0.165	0.224	0.997
		Model 2	0.709 (0.698, 0.720)	0.641 (0.559, 0.723)	0.681 (0.604, 0.759)	0.107	0.440	0.485
	Enhanced	Model 1	0.580 (0.549, 0.610)	0.550 (0.470, 0.629)	0.539 (0.447, 0.631)	0.492	0.411	0.860
		Model 2	0.717 (0.689, 0.745)	0.660 (0.577, 0.743)	0.673 (0.592, 0.754)	0.203	0.321	0.821

For binary outcomes, logistic regressions were used to test for the associations between the PGSs as the independent variables and their corresponding cardiometabolic outcomes as the dependent variables.  Model 1 was unadjusted to obtain crude estimates as the relationship between genotype and phenotype should be unconfounded. Model 2 was adjusted for age, sex, and SEP to obtain more accurate and precise effect size estimates. The classification performance of the logistic regression models was evaluated using the area under the receiver operating characteristic curve. When comparing two AUCs, the p-values were derived using DeLong’s test .Significant *p*-values are highlighted in bold.

*AUC* area under the receiver operating characteristic curve. Other abbreviations as in Table 2.

*White Europeans were compared with South Asians.

**White Europeans were compared with African Caribbeans.

***South Asians were compared with African Caribbeans.

### Type 2 diabetes

According to the OR, the performance was highest in White Europeans (OR 2.48 95% CI [2.39, 2.58]) followed by South Asians (OR 2.05 95% CI [1.91, 2.20]) and African Caribbeans (OR 1.51 95% CI 1.51 [1.30, 1.48]) (Fig. 1B). According to the AUC, the enhanced PGS’ predictive performance was higher in White Europeans (AUC 0.80 95% CI [0.79, 0.82]) compared to South Asians (AUC 0.76 95% CI [0.73, 0.78]) and African Caribbeans (AUC 0.73 95% CI [0.69, 0.76]) (Table 3).

### HbA1c

The regression coefficient (β) was higher in White Europeans and South Asians compared to African Caribbeans. One unit (or 1 SD) increase in the enhanced PGS was associated with a 1.69 mmol/mol 95% CI (1.65, 1.73) higher HbA1c in White Europeans, 1.79 95% CI (1.57, 2.00) in South Asians and 1.03 (0.81, 1.26) in African Caribbeans after adjusting for sex, age, SEP (Table 2). The difference between White Europeans and South Asians was not statistically significant (*p* = 0.370). Results are visually depicted in Fig. 1C.

### BMI

A unit increase in the enhanced PGS resulted in a 1.71 kg/m^2^ 95% CI (1.68, 1.74) increase in BMI in White Europeans, 1.31 95% CI (1.22, 1.40) in South Asians and 0.90 95% CI (0.80, 1.00) in African Caribbeans (Table 2 and Fig. 1D).

### Hypertension

**Fig. 2:**
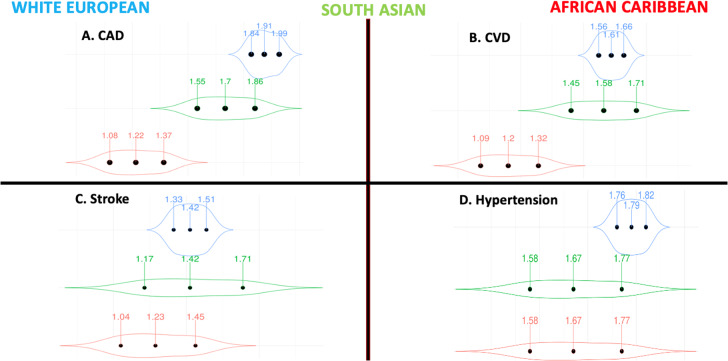
Violin plots highlighting the effect sizes per standard deviation increase in the enhanced PGSs for vascular traits stratified by ethnicity. Our vascular traits included: **(A)** CAD, **(B)** CVD, **(C)** stroke, and **(D)** hypertension. Logistic regressions were used to test for the associations between the PGSs as the independent variables and their corresponding cardiometabolic outcomes as the dependent variables. Models were adjusted for age, sex and SEP. In each violin plot, the middle dot represents the odds ratio, and the adjacent ones the lower and upper limits of the 95% CI. The violin shape reflects the wideness of the 95% CI. **Abbreviations**: CAD coronary artery disease, CI confidence interval, CVD cardiovascular disease, PGS polygenic score, OR odds ratio, SEP socio-economic position.

There was no difference in performance by ethnicity according to the ROC curve analysis (Table 3). The ORs were similar across all ethnicities using both standard (≈1.50) and enhanced PGSs (≈1.70). (Table 2 and Fig. 2C).

### CVD and CAD

For CVD, the performance of the enhanced PGS was higher in White Europeans (AUC 0.77 95% CI [0.76, 0.78]) and South Asians (AUC 0.74 95% CI [0.70, 0.77]) vs African Caribbeans (AUC 0.66 95% CI [0.61, 0.72]) all *p* < 0.025 (Table 3). Similarly, the ORs were higher in White Europeans (OR 1.61 95% CI [1.56, 1.66]) and South Asians (OR 1.58 95% CI [1.45, 1.71]) compared to African Caribbeans (OR 1.20 95% CI [1.09, 1.32]) (Fig. 2A).

For CAD, the results were similar to those reported above for CVD, with a higher predictive performance according to the ROC curve analysis (Table 3) and higher ORs (Table 2) in White Europeans and South Asians compared to African Caribbeans.

### Stroke

The performance of the enhanced PGS according to the ROC curve analysis (AUC ≈ 0.70) and the ORs (1.20–1.40) were similar across all ethnicities (Fig. 2D, Table 3).

### HDL and LDL

**Fig. 3:**
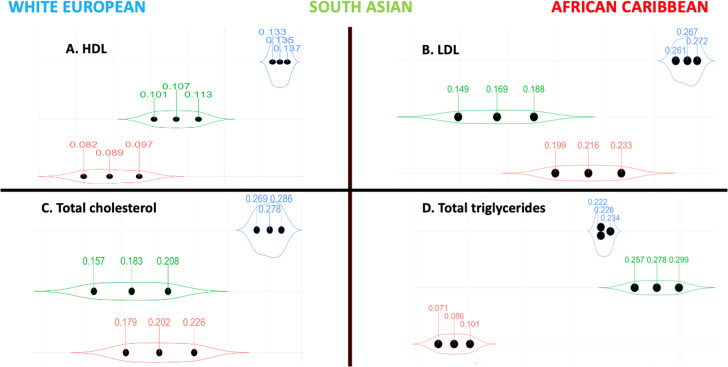
Violin plots highlighting the effect sizes per standard deviation increase in the enhanced PGSs for lipid traits stratified by ethnicity. Our lipid traits included: **(A)** HDL, **(B)** LDL, **(C)** total cholesterol, and **(D)** total triglycerides. Glms with gamma distribution were used to test for the associations between the PGSs as the independent variables and their corresponding cardiometabolic outcomes as the dependent variables. Models were adjusted for age, sex and SEP. In each violin plot, the middle dot represents the β regression coefficient, and the adjacent ones the lower and upper limits of the 95% CI. The violin shape reflects the wideness of the 95% CI. **Abbreviations**: CI confidence interval, glm generalised linear model, HDL high-density lipoproteins, LDL low-density lipoproteins,  PGS polygenic score, SEP socio-economic position.

One SD increase in the enhanced HDL PGS resulted in a 0.135 mmol/l 95% CI (0.133,0.137) greater HDL in White Europeans, 0.107 95% CI (0.101, 0.113) in South Asians and 0.089 95% CI (0.082, 0.097) in African Caribbeans (Table 2 and Fig. 3A, B) after adjusting for sex, age, SEP. A unit increase in the enhanced LDL PGS was associated with a higher LDL in White Europeans (0.267 mmol/l 95% CI [0.261, 0.272]) followed by African Caribbeans (0.216 95% CI [0.199, 0.233]) and South Asians (0.169 95% CI [0.149, 0.188]).

### Total cholesterol and triglycerides

A unit (or 1 SD) increase in the enhanced PGS resulted in a greater total cholesterol in White Europeans (0.278 mmol/l 95% CI [0.269,0.286]) compared to African Carribeans (0.202 95% CI [0.179, 0.226]) and South Aasians (0.183 95% CI [0.157, 0.208]) (Fig. 3C). On the other hand, a unit (or 1 SD) increase in the TTG enhanced PGS was associated with a higher TTG in South Asians (0.278 mmol/l 95% CI [0.257, 0.299]) compared to White Europeans (0.228 95% CI [0.222, 0.234]) and African Caribbeans (0.086 95% CI [0.071, 0.101]) (Fig. 3D).

### Sensitivity analyses

The regression results stratified per ethnicity for HbA1c, HDL, LDL, total cholesterol, and triglycerides with further adjustments for diabetes medications or lipid-lowering drugs as appropriate are presented in Supplementary Table S2. In general, the findings were replicated, but the effect sizes were slightly smaller for HbA1c and slightly larger for the lipid outcomes.

The PR-AUC results stratified by ethnicity are presented in Supplementary Table S3. The PR-AUC was larger in White Europeans followed by South Asians and African Caribbeans for T1DM, CVD and CAD. However, the estimates were similar for hypertension and stroke. The PR-AUC was greater in South Asians compared to White Europeans for T2DM.

## Discussion

In this study we evaluated the performance of standard and enhanced UKB cardiometabolic PGSs derived mostly in White European populations in association with their respective observed phenotype by ethnicity. Whilst the UKB PGSs included some data from multi-ethnic GWAS studies, the performance of both the standard and enhanced PGSs was better in White Europeans compared to South Asians and African Caribbeans for most cardiometabolic outcomes. This can be explained by the predominance of White European GWAS data when deriving the PGSs.

### Factors driving poorer PGS performance in ethnic diverse populations

According to the National Human Genome Research Institute and European Bioinformatics Institute GWAS catalogue almost 80% of the GWAS studies  were performed in White Europeans which represent roughly 10% of the global population [34].  In contrast, 25% of the global population is of South Asian and 15% of African Caribbean ancestry. Thus, GWAS data is scarce in non-White ancestries. This has multiple downstream implications and might partly explain the worse performance of the  PGSs in multi-ethnic populations [35]. Firstly, linkage disequilibrium (LD) varies across ancestries  which may drive differences  in effect size estimates in GWASs [36]. Secondly, imputation reference panels which are widely used to address bias in GWASs are less efficient in non-White ancestries due to data scarcity. Thirdly, within-ethnicity ancestry subcategories in non-White population are less studied. This is important because within-ethnicity heterogeneity leading to differential predictive power of PGSs in the same ethnicity has been reported [11]. Fourthly, the normal reference ranges for quantitative biomarkers may vary between ethnicities [37]. Without ethnicity specific cut-offs, there is an inherent bias in any GWAS which categorises/binarizes quantitative traits. Lastly, studies may be reporting common benign variants as pathologic in other ethnicities just because they are rare in White  Europeans [2]. Thus, large ethnic diverse datasets and improved treatment of LD and variant frequencies are increasingly needed to create equitable PGSs before widespread clinical use [35].

### Ethnic inclusivity for equitable implementation of polygenic scores

In CVD research, the vast majority of cohort studies enroled mostly people of White European ancestry. There are only a few studies which include genetic data in ethnic minorities. These either focus on a single ethnic group (e.g., East London Genes & Health [ELGH], China Kadoorie Biobank [CKB], Mexico City Prospective Study [MCPS], New Delhi Birth Cohort Study, OLA etc.) or multiple ethnic groups (e.g., Age, Gene/Environment Susceptibility-Reykjavik Study [AGES-Reykjavik], ARIC, Born in Bradford (BiB), Cardiovascular Health Study [CHS], Dallas Heart Study [DHS], Framingham Heart Study [FHS] OMNI cohorts, JHS, MEC, MESA, Rotterdam Study [RS], Southall and Brent Revisited [SABRE] etc.). Importantly, there is a tendency to aggregate individual cohorts into consortia (e.g., genetic data from AGES, ARIC, CHS, FHS and RS cohorts are available through the Cohorts for Heart and Aging Research in Genomic Epidemiology [CHARGE] consortium). Despite these collections, the percentage of non-White European ancestry participants in GWASs has not increased in recent years [34]. This suggests that the reduced performance of PGSs in ethnic minorities is unlikely to improve in the near future.

In developed nations, the low participation of ethnic minorities in biomedical research is multi-factorial but mainly related to reduced trust given past research misconduct and feelings of racial discrimination [38]. However, movements such as the All of Us Research Program from National Institute of Health are working towards having a culturally aware approach to engage under-represented ethnic minorities in research [39].

### Ancestry inclusivity for equitable implementation of polygenic scores

Race and ethnicity are socio-cultural constructs, whilst ancestry refers to the genetic origin of a population. Engaging under-represented ethnic and ancestry minorities in genomics research should be a global research priority. Indeed, there are movements aiming to address these disparities such as the Human Heredity and Health in Africa initiative [40]. However, lack of funding remains the main limitation of such international movements [41].

### Polygenic scores and health inequalities during translation to practice

The advent of genetic data in large cohort datasets such as the UK Biobank has led to the discovery of multiple SNPs which are associated with a variety of cardiometabolic diseases using GWASs. Whilst the added value of PGSs on top of already validated clinical tools is yet to be fully elucidated, current studies suggest that PGSs could: (1) increase disease prediction in early life, (2) help guide population-wide screening and preventative targeted interventions (e.g., lipid lowering drugs in those with a high PGS for total cholesterol and LDL), (3) help promote favourable health behaviours in those with an enhanced risk, (4) improve diagnostic accuracy (e.g., differentiating T1DM vs T2DM in overweight antibody-negative young individuals), and (5) predicting response to treatments [27]. Given the worse performance of PGSs in ethnic minorities, they may miss out on benefiting from improved health outcomes. The deployment of PGSs would benefit the population group which is already privileged in terms of health outcomes further deepening existing healthcare inequalities. Thus, large-scale multi-ancestry GWAS data are urgently needed to generate ethnicity stratified PGSs to tackle health inequalities.

### Limitations

Limitations of the UK Biobank PGSs have been previously discussed [26]. With regards to PGS evaluation, the main limitation of our study relates to the lack of widely accepted performance metrics [42]. Whilst phenotypic variance explained (R^2^) and association *p*-values have been previously proposed [43], we used effect-size metrics for the outcome as these are widely used for established traditional risk factors. However, these do not accurately capture disease prevalence in the general population.

In addition, the use of the self-reported ethnicity is not in line with the latest recommendations on the use and reporting of race and ancestry in genetic research, which instead recommends the incorporation of ancestry informative markers for a more precise characterisation of an individual’s identity [5, 44]. Importantly, whilst there is a good overlap between genetic ancestry and self-reported ethnicity, these are not identical in the UK Biobank [25]. Nonetheless, self-reported ethnicity is also able to capture social constructs (e.g., health beliefs) which could drive the observed differences in PGSs’ performance. In addition, detailed ethnic groupings (defined according to the 16 categories of the 2001 census) had to be collapsed into the 5 high level categories of the census to increase the statistical power of the analyses conducted in multi-ethnic populations.

As underlying differences in allele frequencies and LD likely make a major contribution to the ancestry performance differences in non-ancestry matched PGSs, the scores could be improved through the use of the appropriate ancestral reference LD. However, we did not attempt to improve the PGSs in this study as our aim was solely to evaluate the ones derived by Thompson et al. [26]. While our findings that PGSs perform better in White Europeans may not be unsurprising, there is a need to provide empirical evidence, as without this, the proposed PGSs will be used without consideration of the ethnic background. While Thompson et al. [26] provided the effect sizes for the associations between PGSs with their corresponding outcomes stratified by ethnicity, we were able to build on this work by using more sophisticated statistical approaches (e.g., ROC and precision recall curves) to better evaluate the performance of the PGSs in multi-ethnic populations. Our work is important because it highlights that these PGSs released by UK Biobank need to be used with consideration in multi-ethnic populations and underscores the need for improving them. In addition, we discuss the factors driving poorer PGS performance in ethnically diverse populations, and the need for ethnic inclusivity for the equitable implementation of PGSs to reduce health inequalities as they transition to clinical practice.

Although the ethnic minorities were 3–4 years younger at recruitment, incident cardiometabolic diseases have a younger age of onset (e.g., diabetes in South Asians [45] and hypertension in those of African ancestry [46] occur ≈10-years earlier compared to White Europeans). In addition, SABRE data showed that a lower proportion of UK White individuals were diagnosed with diabetes in the National Health Service compared to South Asians and African Caribbeans when comparing the study blood test results with the healthcare records [47]. As a higher proportion in the ethnic minority groups should have already been diagnosed with the cardiometabolic diseases, it would be expected that PGSs would actually perform better in the ethnic minorities. Thus, another important limitation is that we might actually underestimate the performance difference of the PGSs between White Europeans and the multi-ethnic populations.

## Conclusion

In general, UK Biobank standard and enhanced PGSs had markedly better performance in White Europeans compared to South Asians and African Caribbeans when evaluating cardiometabolic phenotypes. More GWAS data in ethnic minorities is required to improve the performance of the PGSs to avoid perpetuating health inequalities especially since cardiometabolic diseases are more prevalent in South Asians and African Caribbeans.

### Supplementary information

Suplementary Material

## Data Availability

The UK Biobank data is available via an application from https://www.ukbiobank.ac.uk/. This work was conducted under application ID 7661.
