# Supplementary material for: Validity of European-centric cardiometabolic polygenic scores in multi-ancestry populations
Source: Eur J Hum Genet. 2024 Jan 5;32(6):697–707. doi: 10.1038/s41431-023-01517-3 (PMC11153583; doi:10.1038/s41431-023-01517-3)
Supplement: Supplementary file 1 — Suplementary Material [file 41431_2023_1517_MOESM1_ESM.docx]

**Supplementary material for:**

**Validity of European-centric cardiometabolic**

**polygenic scores in multi-ancestry populations**

**Authors:** Constantin-Cristian Topriceanu^1,2^, Nish Chaturvedi^1,2^, Rohini Mathur^3*^, Victoria Garfield^1,2*^

*Joint last authors

**Author Affiliations:**

1. Department of Population Science and Experimental Medicine, Institute of Cardiovascular Science, University College London, Gower Street, London WC1E 6BT, UK.
2. MRC Unit for Lifelong Health and Ageing, University College London, 1-19 Torrington Place, London WC1E 7HB, UK
3. Centre for Primary Care, Wolfson Institute of Population Health, Queen Mary University of London, London, UK

**Corresponding author**

Dr. Constantin-Cristian TOPRICEANU

Research Fellow, UCL MRC Unit for Lifelong Health and Aging

1-19 Torrington Place, London, WC1E 7HB

Email: [zchatop@ucl.ac.uk](mailto:zchatop@ucl.ac.uk)

**Supplementary Table S1. Enhanced polygenic scores per ethnicity in UK Biobank.**

|  | **White European [1]** | | **South Asian [2]** | | **African Caribbean [3]** | | **[1] vs [2]** | **[1] vs [3]** | **[2] vs [3]** |
| --- | --- | --- | --- | --- | --- | --- | --- | --- | --- |
|  | **n** | **Count (%) or Mean± sd** | **n** | **Count (%) or Mean± sd** | **n** | **Count (%) or Mean± sd** | **p-value*** | **p-value**** | **p-value***** |
| **Polygenic scores** | | | | | | | | | |
| T1DM | 76877 | 0.00 ± 1.11 | 7637 | -0.09 ± 1.04 | 7618 | 0.00 ± 1.11 | **<0.0001** | 0.883 | **<0.0001** |
| T2DM | 76877 | -0.12 ± 1.04 | 7637 | 0.03 ± 1.02 | 7618 | 0.04 ± 1.16 | **<0.0001** | **<0.0001** | 0.351 |
| HbA1c | 76877 | -0.05 ± 1.09 | 7637 | 0.11± 1.10 | 7618 | -0.11 ± 1.11 | **<0.0001** | **<0.0001** | **<0.0001** |
| BMI | 76877 | -0.01 ± 1.06 | 7637 | -0.09 ± 1.06 | 7618 | -0.07 ± 1.14 | **<0.0001** | **<0.0001** | 0.231 |
| Hypertension | 76877 | -0.02 ± 1.01 | 7637 | -0.17 ± 1.02 | 7618 | -0.13 ± 1.11 | **<0.0001** | **<0.0001** | **0.017** |
| CVD | 76877 | -0.08 ± 1.04 | 7637 | 0.05 ± 1.03 | 7618 | -0.17 ± 1.12 | **<0.0001** | **<0.0001** | **<0.0001** |
| CAD | 76877 | -0.12 ± 1.02 | 7637 | -0.02 ± 1.07 | 7618 | -0.18 ± 1.13 | **<0.0001** | **<0.0001** | **<0.0001** |
| Stroke | 76877 | 0.01 ± 0.99 | 7637 | -0.09 ± 1.00 | 7618 | -0.22 ± 1.16 | **<0.0001** | **<0.0001** | **<0.0001** |
| HDL | 76877 | -0.02 ± 1.07 | 7637 | 0.16 ± 1.06 | 7618 | 0.01 ± 1.08 | **<0.0001** | **0.042** | **<0.0001** |
| LDL | 76877 | -0.01 ± 1.08 | 7637 | 0.19 ± 1.03 | 7618 | -0.09 ± 1.09 | **<0.0001** | **<0.0001** | **<0.0001** |
| Total cholesterol | 76877 | -0.07 ± 1.03 | 7637 | 0.17 ± 1.01 | 7618 | -0.07 ± 1.08 | **<0.0001** | 0.760 | **<0.0001** |
| TTG | 76877 | -0.07 ± 1.03 | 7644 | 0.17 ± 1.01 | 7618 | -0.07 ± 1.07 | **<0.0001** | 0.760 | **<0.0001** |

*BMI = body mass index; CAD = coronary artery disease; HbA1c = glycated hemoglobin A1c; CVD = cardiovascular disease; HDL = high-density lipoproteins; LDL = low-density lipoproteins; sd = standard deviation; SEP = socio-economic position; T1DM = type 1 diabetes mellitus; T2DM = type 2 diabetes mellitus; TTG = total triglycerides.*

All p-values were derived using t-test. Significant p-values are presented in bold.

*White Europeans were compared with South Asians

**White Europeans were compared with African Caribbeans.

***South Asians were compared with African Caribbeans.

**Supplementary Table S2. Regression results stratified per ethnicity with and without adjustment for medications.**

|  |  |  | **White European** | | **South Asian** | | **African Caribbean** | |
| --- | --- | --- | --- | --- | --- | --- | --- | --- |
| **Outcome** | **PGS type** | **Model** | **β (95% CI)** | **p-value** | **β (95% CI)** | **p-value** | **β (95% CI)** | **p-value** |
| HbA1c | Standard | Model 2^a^ | 0.83 (0.81, 0.85) | **<0.0001** | 1.28 (1.04, 1.52) | **<0.0001** | 0.51 (0.28, 0.73) | **<0.0001** |
|  |  | Model 2^b^ | 0.64 (0.62, 0.65) | **<0.0001** | 0.82 (0.64,0.99) | **<0.0001** | 0.45 (0.28, 0.62) | **<0.0001** |
|  | Enhanced | Model 2^a^ | 1.69 (1.65, 1.73) | **<0.0001** | 1.79 (1.57, 2.00) | **<0.0001** | 1.03 (0.81, 1.26) | **<0.0001** |
|  |  | Model 2^b^ | 1.53 (1.50, 1.57) | **<0.0001** | 1.30 (1.14, 1.46) | **<0.0001** | 0.91 (0.75, 1.08) | **<0.0001** |
| HDL | Standard | Model 2^a^ | 0.13 (0.13, 0.14) | **<0.0001** | 0.11 (0.10, 0.12) | **<0.0001** | 0.10 (0.09, 0.11) | **<0.0001** |
|  |  | Model 2^b^ | 0.13 (0.13, 0.13) | **<0.0001** | 0.11 (0.10, 0.12) | **<0.0001** | 0.10 (0.09, 0.11) | **<0.0001** |
|  | Enhanced | Model 2^a^ | 0.14 (0.13, 0.14) | **<0.0001** | 0.11 (0.10, 0.12) | **<0.0001** | 0.09 (0.08, 0.10) | **<0.0001** |
|  |  | Model 2^b^ | 0.13 (0.13, 0.14) | **<0.0001** | 0.11 (0.10, 0.11) | **<0.0001** | 0.09 (0.08, 0.10) | **<0.0001** |
| LDL | Standard | Model 2^a^ | 0.24 (0.23, 0.24) | **<0.0001** | 0.15 (0.13, 0.17) | **<0.0001** | 0.15 (0.14, 0.17) | **<0.0001** |
|  |  | Model 2^b^ | 0.27 (0.27, 0.28) | **<0.0001** | 0.19 (0.17, 0.20) | **<0.0001** | 0.17 (0.15, 0.19) | **<0.0001** |
|  | Enhanced | Model 2^a^ | 0.27 (0.26, 0.27) | **<0.0001** | 0.17 (0.15, 0.19) | **<0.0001** | 0.22 (0.20, 0.23) | **<0.0001** |
|  |  | Model 2^b^ | 0.30 (0.30, 0.31) | **<0.0001** | 0.22 (0.20, 0.23) | **<0.0001** | 0.24 (0.22, 0.26) | **<0.0001** |
| Total cholesterol | Enhanced | Model 2^a^ | 0.28 (0.27, 0.29) | **<0.0001** | 0.18 (0.16, 0.21) | **<0.0001** | 0.20 (0.18, 0.23) | **<0.0001** |
|  |  | Model 2^b^ | 0.31 (0.30, 0.32) | **<0.0001** | 0.22 (0.19, 0.24) | **<0.0001** | 0.22 (0.20, 0.24) | **<0.0001** |
| TTG | Enhanced | Model 2^a^ | 0.23 (0.22, 0.24) | **<0.0001** | 0.27 (0.26, 0.30) | **<0.0001** | 0.09 (0.07, 0.10) | **<0.0001** |
|  |  | Model 2^b^ | 0.23 (0.22, 0.23) | **<0.0001** | 0.28 (0.25, 0.30) | **<0.0001** | 0.09 (0.07, 0.10) | **<0.0001** |

Generalised linear models with gamma distribution and identity links were used to test for the associations between the PGSs as the independent variables and their corresponding cardiometabolic outcomes as the dependent variables. Effect sizes are presented in the form of β regression coefficients. Model 1 was unadjusted to obtain crude estimates as the relationship between genotype and phenotype should be unconfounded. Model 2^a^ was adjusted for age, sex, and SEP to obtain more accurate and precise effect size regression estimates. Model 2^b^ for HbA1c was in addition adjusted for diabetes medications, while HDL, LDL, total cholesterol and TTG models were adjusted for lipid-lowering medications. Significant p-values are highlighted in bold.

*β = regression coefficient; CI = confidence interval; SEP = socio-economic position.* Other abbreviations as in **Supplementary Table S1**.

**Supplementary Table S3. Predictive power of PGSs for cardiometabolic binary outcomes stratified by ancestry.**

| **Outcome** | **PGS** | **Model** | **White European** | **South Asian** | **African Caribbean** |
| --- | --- | --- | --- | --- | --- |
|  |  |  | **PR-AUC** | **PR-AUC** | **PR-AUC** |
| T1DM | Standard | Model 1 | 0.033 | 0.011 | 0.018 |
|  |  | Model 2 | 0.038 | 0.009 | 0.005 |
|  | Enhanced | Model 1 | 0.033 | 0.012 | 0.018 |
|  |  | Model 2 | 0.040 | 0.009 | 0.006 |
| T2DM | Standard | Model 1 | 0.108 | 0.281 | 0.168 |
|  |  | Model 2 | 0.156 | 0.355 | 0.255 |
|  | Enhanced | Model 1 | 0.117 | 0.287 | 0.158 |
|  |  | Model 2 | 0.168 | 0.365 | 0.239 |
| Hypertension | Standard | Model 1 | 0.734 | 0.754 | 0.753 |
|  |  | Model 2 | 0.789 | 0.823 | 0.847 |
|  | Enhanced | Model 1 | 0.739 | 0.771 | 0.775 |
|  |  | Model 2 | 0.790 | 0.832 | 0.854 |
| CVD | Standard | Model 1 | 0.124 | 0.102 | 0.006 |
|  |  | Model 2 | 0.247 | 0.197 | 0.083 |
|  | Enhanced | Model 1 | 0.132 | 0.110 | 0.063 |
|  |  | Model 2 | 0.256 | 0.201 | 0.086 |
| CAD | Standard | Model 1 | 0.115 | 0.075 | 0.038 |
|  |  | Model 2 | 0.190 | 0.159 | 0.058 |
|  | Enhanced | Model 1 | 0.125 | 0.087 | 0.041 |
|  |  | Model 2 | 0.206 | 0.174 | 0.057 |
| Stroke | Standard | Model 1 | 0.021 | 0.022 | 0.019 |
|  |  | Model 2 | 0.038 | 0.042 | 0.029 |
|  | Enhanced | Model 1 | 0.020 | 0.024 | 0.044 |
|  |  | Model 2 | 0.040 | 0.046 | 0.032 |

For binary outcomes, logistic regressions were used to test for the associations between the PGSs as the independent variables and their corresponding cardiometabolic outcomes as the dependent variables.  Model 1 was unadjusted to obtain crude estimates as the relationship between genotype and phenotype should be unconfounded. Model 2 was adjusted for age, sex, and SEP to obtain more accurate and precise effect size regression estimates. The classification performance of the logistic regression models was evaluated using the PR-AUC.

*PR-AUC = area under the precision-recall curve*. Other abbreviations as in **Supplementary Table S2**.
